# A rare variation of ERCC8 gene cause Cockayne syndrome in a Chinese family

**DOI:** 10.3389/fgene.2025.1531832

**Published:** 2025-03-12

**Authors:** Fengjuan Ding, Fei Hou, Bowen Zhao, Hua Jin

**Affiliations:** Department of Prenatal Diagnosis, Jinan Maternity and Child Care Hospital Affiliated Shandong First Medical University, Jinan, Shandong, China

**Keywords:** Cockayne syndrome, medical exome sequencing, ERCC8, prenatal diagnosis, rare inherited disorders

## Abstract

**Background:**

Cockayne syndrome (CS) is a multisystem degenerative disorder in which dysplasia and microcephaly represent the primary criteria for diagnosis. we present the cases of two patients who exhibited distinctive facial features and a range of other clinical manifestations, including growth failure, developmental delay, microcephaly, dental anomalies, and unstable gait.

**Methods:**

Clinical information pertaining to the patient’s family was collated and the Pedigree chart was drawn. Two milliliters of peripheral blood were drawn from each of the two patients (III1and III3) and their parents, The causative genes were identified by Medical exome sequencing. Furthermore, the pregnant women underwent amniotic fluid prenatal diagnosis at mid-pregnancy (III5).

**Results:**

Medical exome sequencing revealed that both patients had a homozygous deletion of Exon4 in the ERCC8 gene and that both parents were carriers. Prenatal diagnosis by amniotic fluid confirmed that the fetus (III5) did not carry the variant.

**Conclusion:**

This clarified the diagnosis at the genetic level, deepened our understanding of the disease, and facilitated the ability to provide accurate genetic counseling and prenatal diagnosis, with the goal of reducing the number of new affected individuals in the family.

## 1 Introduction

Cockayne Syndrome (CS) is a rare and fatal neurodegenerative disorder with autosomal recessive inheritance, characterized by growth retardation, neurodevelopmental deficits, and premature aging (Kraemer et al., 2007). Statistical data indicates that the prevalence of CS in the United States is approximately 2.5/1,000,000, with an average age of death of 12 years (Karikkineth et al., 2017). Cockayne syndrome is classified into three distinct types, designated as Type I, Type II, and Type III, based on clinical presentation and severity. CS is genetically heterogeneous, and it is established that pathogenic variants in the XPD, XPG, XPB, ERCC6, and ERCC8 genes can cause CS. Additionally, it is known that 90% of the causative genes are ERCC8 (CS type I) and ERCC6 (CS type II). Of these, mutations in the CS type I gene account for approximately one-third of cases, while mutations in the CS type II gene account for approximately two-thirds (Laugel, 2013).

The early diagnosis of CS is primarily dependent on the patient’s clinical presentation and cranial imaging changes. However, due to the similarities in clinical manifestations with other central nervous system degenerative diseases, it can be challenging to make an accurate diagnosis. Fetuses typically develop normally in the prenatal stage, exhibiting no discernible indications for prenatal diagnosis. Moreover, patients in the early stages of the disease do not manifest notable features of prematurity, rendering the identification of genes associated with CS a challenging endeavor. The utilization of the WES test is further complicated by the fact that CNVs can be easily overlooked by exome sequencing, particularly when the quality of the DNA in the amniotic fluid is suboptimal in prenatal diagnosis. In this study, a high-precision medical exome sequecing test and following qPCR demonstrated that the proband and his cousin were CSA patients, resulting from a homozygous deletion of the Exon4 in the ERCC8 gene. Furthermore, both parents were identified as heterozygous carriers of this CNV. The proband’s mother underwent prenatal diagnosis of the amniotic fluid to ascertain the likelihood of a healthy birth. exome sequencing of aminotic fluid revealed the presence of a heterozygous deletion of the Exon4 gene in the ERCC8 gene of the fetus. The findings of our study elucidate the genetic etiology of the patients, increase the number of clinical cases, deepen our understanding of the disease, and provide a theoretical basis and guidance for reproductive genetic counseling and prenatal diagnosis for families.

## 2 Materials and methods

### 2.1 Patients and clinical information

**FIGURE 1:**
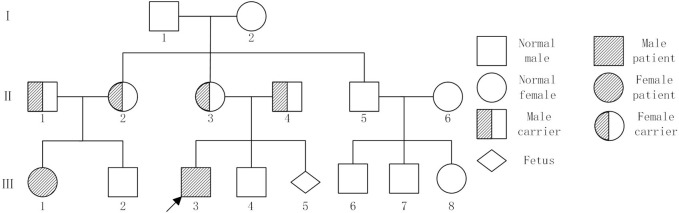
Pedigree chart showed individuals with or without Cockayne syndrome. Family members II1, II2, II3,II4, III1, III3 and III5 underwent testing. Note: I, II, and III represent the generations. Squares represent males, large circles represent females, solid black symbols indicate affected patients,half-solid black symbols indicate carriers,the arrow indicates the proband.

The male proband (III3), aged seven, was admitted to the prenatal diagnostic centre of Jinan Maternal and Child Health Hospital due to concerns regarding developmental delay and growth retardation. A pedigree chart was constructed by tracing the family members of the first witness back to their relatives (Figure 1). This family was comprised three generations, with a total of two patients (III1 and III3). Both patients exhibited a constellation of symptoms, including developmental delay, growth retardation, photosensitivity, premature aging, gait abnormalities, and the presence of neurodegenerative lesions (Table 1). The mother of the first witness underwent genetic counselling and requested an amniotic fluid prenatal diagnosis at our prenatal diagnostic centre with the objective of ensuring the healthy development of the foetus. The study was approved by the Ethics Committee of Jinan Maternal and Child Health Hospital Approval No. (No. KY R24-039) in accordance with the principles set forth in the Declaration of Helsinki. Following the acquisition of written and signed informed consent from patients and their families, samples and information were collected. It should be noted that all patient data was used anonymously.

**TABLE 1:** Clinical information for the two patients.

Patient ID	III3	III1
Gender	Male	Female
Age of diagnosis	7	15
Growth parameters		
Height	-3SD	-3SD
Weight	-3SD	-3SD
Facial features		
Microcephaly	-3SD	-3SD
Sunken eyes	+	+
Pointed nose	+	+
Micrognathia	+	+
Large auricle	+	+
Dental caries	+	+
Photosensitivity	+	+
Mental retardation	+	+
Kyphosis	+	+
Restricted motion of joints	+	+
Hearing loss	+	+
Leukodystrophy	+	unknown

**FIGURE 2:**
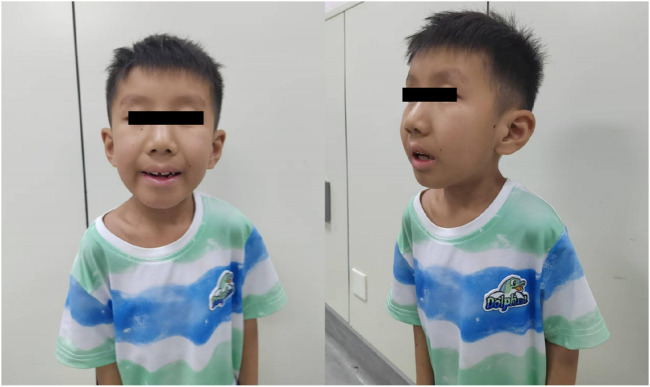
Clinical characteristics of the proband(III3).

III3: The proband was a full-term normal birth in 2017 with a birth weight of 2250 g. The postnatal response was unremarkable, while breastfeeding proved challenging. The infant demonstrated typical development up to 6 months of age, attained sitting at 8 months, and exhibited crawling at over 1 year of age. At around 1.5 years of age, the infant began to walk with support. A cranial magnetic resonance examination of an 11-month-old child revealed a slight delay in the myelination of the white matter and the anterior border of the internal capsule of the brain on both sides. The family expressed concern regarding the efficacy of the rehabilitation therapy, citing suboptimal outcomes. They also highlighted the patient’s susceptibility to sunburn and the gradual decline in his hearing. The physical examination revealed the following: height = 98 cm, weight = 13 kg, head circumference = 48 cm, unresponsive, dry and rough skin, sunken eye sockets, high nose and micrognathia (Figure 2). It is possible to communicate in a straightforward manner despite the presence of slurred speech. The subject displays poor fine hand movements and coordination. The subject exhibited a tendency to walk with both feet turned outwards, along with a high muscle tone in both lower limbs and limited ankle joint movement. The results of the cardiopulmonary and abdominal examinations were normal.

**FIGURE 3:**
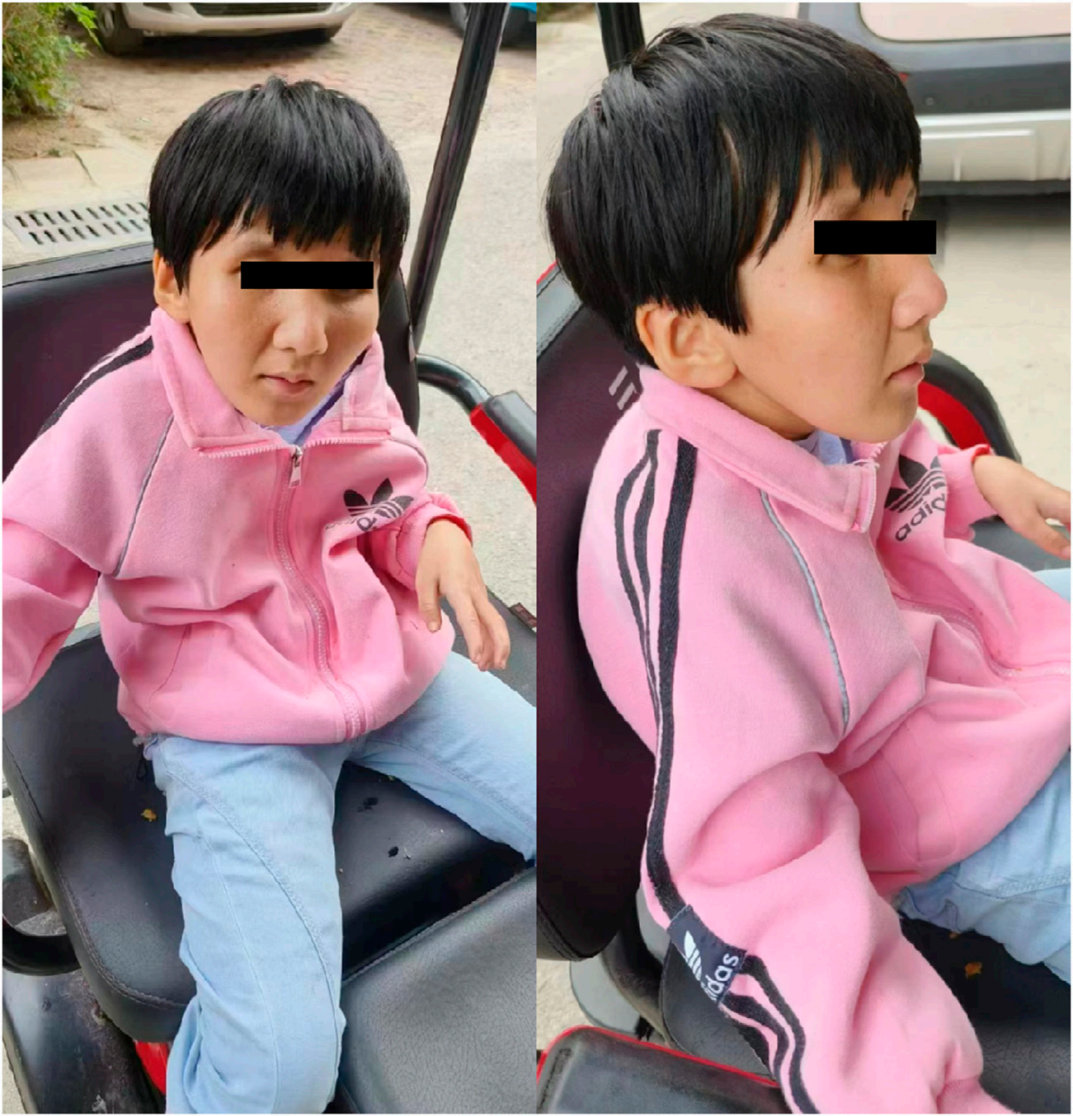
Clinical characteristics of the patient(III1).

III1: The patient is a 15-year-old female with growth retardation and malignant dwarfism. The physical examination revealed the following: height = 112 cm, weight = 25 kg, special facial features (specific eye socket depression, pointed nose, micrognathia, large auricle), dental caries, light sensitivity, limited joint movement, gait abnormality, and so on (Figure 3).

### 2.2 Medical exome sequencing (MES)

After signing the informed consent, peripheral blood samples of specific family members (II1, II2, II3, II4 and III3)and amniotic fluid sample of the proband’s mother (III5) were collected for genomic DNA extraction. Following the standard procedure for a DNA library construction, exome targets were captured using a custom-designed medical exome capture kit (AmCare Genomic Lab, Guangzhou, China). The kit specifically covers the coding region of approximately 4,000 morbid genes that correspond to human genetic diseases as listed in the Online Mendelian Inheritance in Man (OMIM) database. Subsequently, the captured libraries were sequenced using the Illumina HiSeq platform (Illumina, Inc., San Diego, CA, United States) to generate raw paired-end reads of 150 base pairs each. qPCR was performed to verify the deletion of Exon4 of ERCC8 gene detected in MES, and that of III1. PCR amplification was conducted using a quantitative polymerase chain reaction (qPCR) approach with a forward primer (5′-GGT​TTA​GGA​TTA​AAT​TCT​CCT​TTA​T-3′) and a reverse primer (5′-TGA​TAA​AAC​TCT​GAA​AGT​ATG​GG-3′). The annealing temperature was set at 63°C, and 32 cycles were performed. The amplified fragment size was 102 base pairs (bp).

### 2.3 Pathogenicity analysis

To retrieve literature related to variants, databases such as HGMD, PubMed, ClinVar, and others were searched. The content of the retrieved literature was then subjected to analysis, with the American College of Medical Genetics and Genomics (ACMG) variant classification guidelines being consulted in order to classify the variants (Richards et al., 2015). The interpretation of the CNV variants was conducted in accordance with the standards and guidelines recommended by the American College of Medical Genetics and Genomics and the Association for Molecular Pathology (Riggs et al., 2020). In the context of pathogenicity analysis of copy number variation (CNV), reference was made to a number of public databases, including DGV (http://dgv.tcag.ca/dgv/app/home), DECIPHER (https://www.deciphergenomics.org/), OMIM (https://www.omim.org/), and ClinGen (https://www.clinicalgenome.org/).

## 3 Results

### 3.1 Clinical analysis

III3: Male, 7 years and 6 months old. He exhibited a gradual decline in developmental milestones. At 11 months of age, a cranial magnetic resonance examination revealed slight delays in the myelination of the bilateral cerebral white matter and internal capsule, anterior limb. The physical examination revealed the following: height = 98 cm, weight = 13 kg, head circumference = 48 cm, unresponsive, dry and rough skin, sunken eye sockets, high nose, micrognathia (Figure 2). He was able to communicate in a basic manner, although his speech was somewhat indistinct. The subject demonstrated poor fine motor control and coordination. The feet were observed to be turned out when walking, there was a high muscle tone in both lower limbs, and there was limited ankle joint movement.

III1: Female, 15 years and 8 months old, who showed growth retardation. Examination: height = 112 cm, weight = 25 Kg, with special facial features such as sunken eye sockets, pointed nose, micrognathia, large auricle and dental caries. Besides, she was found with light sensitivity, limited joint movement, and gait abnormality (Figure 3).

Both patients in this family lineage met the diagnostic criteria for CS (Laugel, 2013).

### 3.2 Genetic analysis of the family

**FIGURE 4:**
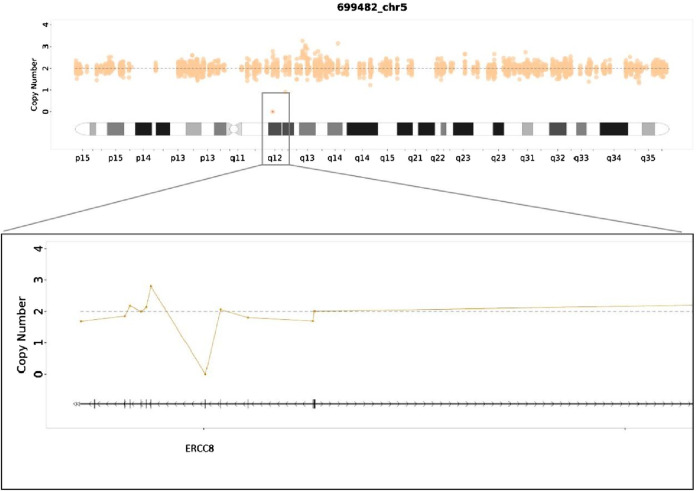
Medical exome sequecing showed a homozygous deletion of Exon4 in ERCC8 gene of the proband.

**FIGURE 5:**
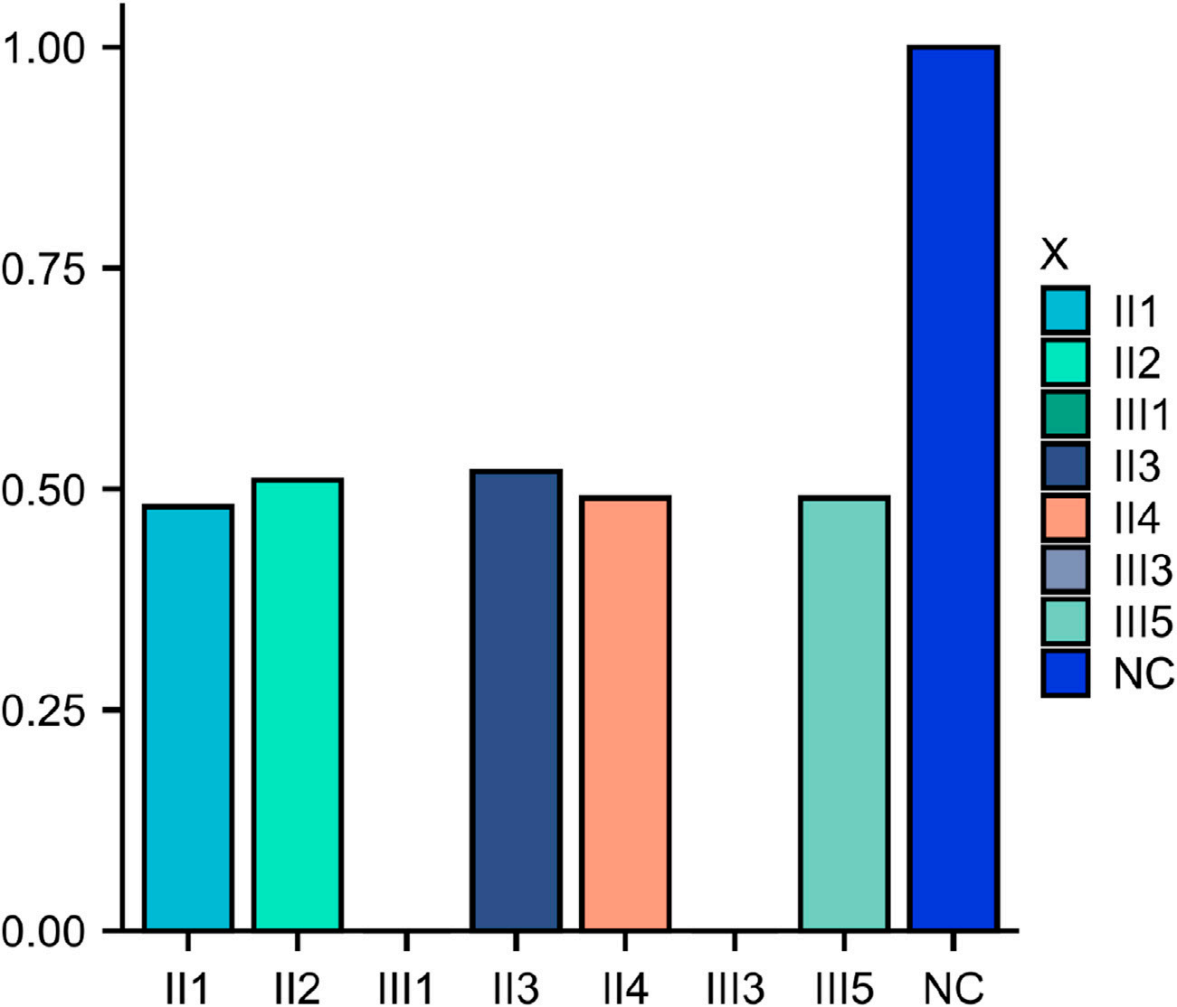
Family members qPCR verification chart.III1 and III3 copy number is 0.

MES was conducted on the proband (III3), and the results indicated a homozygous deletion of Exon4 of the ERCC8 gene (Figure 4). Both parents were identified as carriers with heterozyous deletion of Exon4 of the ERCC8 gene. The proband exhibited characteristics consistent with the genetic variant of CSA, and the result was verified to be reliable through qPCR. III1 also validated as a homozygous deletion of Exon4 in the ERCC8 gene. A prenatal diagnosis of maternal amniotic fluid revealed that the CMA-seq (Chromosome Analysis by Medium Coverage Whole Genome Sequencing) (Lü et al., 2023). results of the fetus (III5) did not indicate the presence of pathogenic chromosomal copy number alterations or AOHs associated with developmental delay. However, MES indicated that the fetus exhibited a heterozygous deletion of the Exon4 of ERCC8 gene, which was subsequently validated through qPCR (Figure 5).

### 3.3 Pathogenicity analysis of the variant

The frequency of deletion of Exon4 in the ERCC8 gene was less than 0.5% in the gnomAD population (PM2). The software predicted that the deletion variant in this gene might affect the reading frame of the gene (PVS1), as the region where the variant is located is an important part. The WD repeat protein is highly conserved across species, as evidenced by the conservation analysis, which also revealed that the amino acid sequences are highly conserved (PP3). According to the ACMG Genetic Variation Classification Criteria and Guidelines, this variant is classified as pathogenic (PM2+PP3+PVS1+PM3).

## 4 Discussion

CS is a rare progressive exacerbation of a multisystem genetic disorder with heterogeneous clinical manifestations. The ERCC8 gene is the common causative gene for the disease. Located on chromosome 5q12.1, the gene encodes a WD-repeat protein that plays a critical role in nucleotide excision repair (NER), particularly in the transcription-coupled repair (TCR) pathway. The deletion of Exon4 is predicted to disrupt the reading frame of the gene (PVS1), leading to a truncated or non-functional protein (Henning et al., 1995). Ultraviolet radiation from the sun, toxic chemicals, and free radicals can all cause damage to DNA. If this damage is not corrected, it can accumulate over time, leading to abnormal cell function and potentially cell death (Berneburg et al., 2000; Cadet et al., 2004). In CS, the loss of function impairs the repair of UV-induced DNA damage and oxidative stress, resulting in the accumulation of DNA lesions, cellular dysfunction, and accelerated aging (Venema et al., 1990). Some studies have indicated that mitochondrial dysfunction, mediated by impaired NAD + signaling and mitophagy, may also contribute to the disease progression in CS patients with ERCC8 mutations. This may involve the impairment of the NAD + mitophagy axis, which could result in increased PARP-1-mediated NAD + consumption and decreased SIRT-1 activity, and subsequently affect mitochondrial homeostasis (Okur et al., 2020). Other studies have indicated that both CS type I and CS type II play a pivotal role in ubiquitin/proteasome-mediated protein degradation, a mechanism that may elucidate the impaired functionality of a multitude of cells in patients with CS and provide novel potential targets for the development of efficacious therapeutic interventions (Paccosi and Proietti-De-Santis, 2021).

**FIGURE 6:**
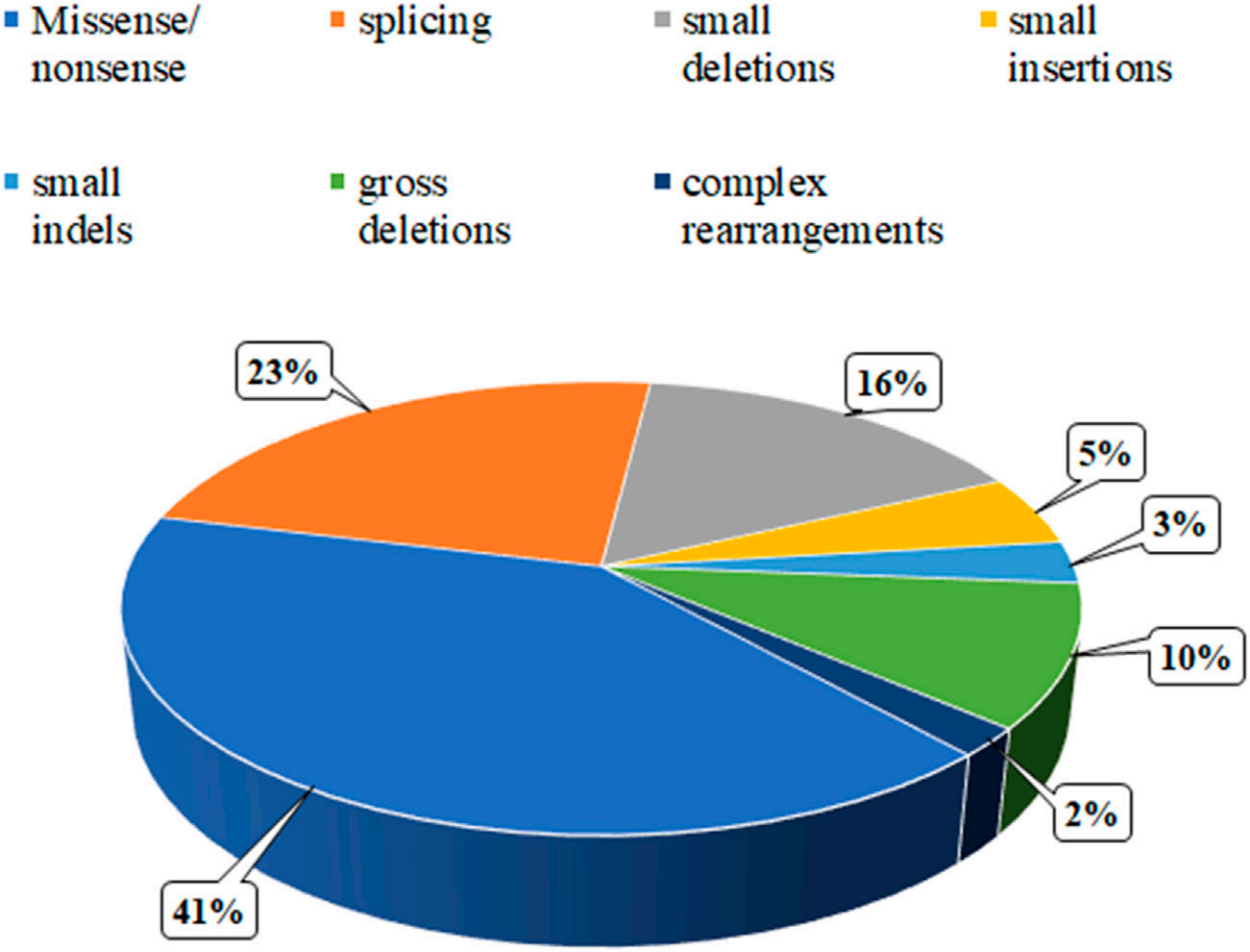
Distribution of the mutation spectrum in ERCC8 gene reported in HGMD. A total of 99 mutations in ERCC8 gene including 41% missense/nonsense mutations, 23% splicing, 16% small deletions, 5% small insertion, 3% small indels, and 10% gross deletions, and 2% complex rearrangements. The mutations in our patients were all documented by HGMD.

To date, the specialized Human Gene Mutation Database (HGMD) has documented 99 variants in the ERCC8 gene, the majority of which are missense or nonsense mutations, with a smaller number of copy number variants (Figure 6). In this case, MES and CMA-seq technology revealed that the proband and his cousin had a heterozygous deletion of Exon4 in the ERCC8 gene, which was consistent with the CS type I genetic variant and compatible with the clinical phenotype of the child. Subsequently, the patient was provided with further genetic counseling and prenatal diagnosis of a fetus carrying a heterozygous deletion of Exon4 in the ERCC8 gene, in addition to both parents being carriers of this CNV. Xie et al. (2017) found a complex rearrangement of Exon4 deletions in the ERCC8 gene, specifically, containing two distinct deletions and an inverted fragment in the middle, the two deletions being chr5:60211534-60213756 (with the removal of Exon4) and chr5:60212086-60217114, with a 1670 bp inverted fragment (chr5:60212086-60213756) with microhomology (TAA and AGCT) around the four breakpoints. This complex rearrangement may be caused by two FoSTeS events, culminating in a deletion/inversion/deletion rearrangement of the ERCC8 region. A comparable complex rearrangement of Exon4 of the ERCC8 gene has also been identified, wherein the deletion encompasses a 1,656 bp intron 4 inverted fragment ([hg18]chr5: 60,247,847-60,249,502) in conjunction with an 8 bp insertion. An analysis of the deletion region reveals the presence of a series of repetitive elements, including LINE, SINE, and LTR elements, among others. The presence of repetitive elements in the region may contribute to its susceptibility to deletions, insertions, and inversions (Ting et al., 2015). The latest literature also identified the same complex rearrangement of Exon4 deletion of the ERCC8 gene as previously observed, thereby supporting the notion of a founder effect in East Asian patients (Watanabe et al., 2024). In this case, the medical exome probe was designed to cover only the coding exon region and not the non-coding region within the rearrangement variant. As a result, only the Exon4 deletion was detected, and the other breakpoint information of the rearrangement could not be identified. Nevertheless, the MES enabled the detection of the 124 bp deletion of Exon4 of the ERCC8 gene in both the amniotic fluid and peripheral blood specimens. This is sufficient for the timely identification of patients and carriers.

While the limited sample size restricts the generalisability of the findings, the identification of a rare homozygous deletion in the ERCC8 gene in two affected individuals from the same family by performing MES provides strong evidence for its pathogenicity. However, it should be noted that MES is not without its limitations, most notably its inability to detect non-coding variants and complex structural rearrangements. Previous studies have demonstrated the high sensitivity and reliability of MES for CNV (Zeng et al., 2024). We also recommend combining MES with complementary techniques (e.g., qPCR, MLPA, or whole-genome sequencing) to improve the detection of such variants in future studies. In families with a history of CS, comprehensive genetic counselling is imperative to elucidate the risks, benefits and limitations of prenatal testing. It is of the utmost imperative to note that long-term monitoring and psychological support are of the utmost importance for families affected by rare genetic diseases.

## 5 Conclusion

In conclusion, using MES, our study confirmed the presence of parent inhereited exon deletion of ERCC8 gene in 2 patients and a fetus in a family. Collected clinical characteristics of patients enriched the clinical spectrum of homozygous deletion of ERCC8 Exon4. Moreover, the amniotic fluid prenatal diagnosis confirmed that the fetus is a carrier of the ERCC8 gene, there by presenting the molecular evidence to the family for counseling. Although the deletion of Exon4 in ERCC8 gene has been identified as a hot spot in asian CS type I patients previously, our study demonstrated the effectiveness of single exon deletion by performing MES in CS type I patients and their families, which emerges MES as an economical and dependable option for the clinical Exon-CNV detection in both prenatal and postnatal samples.

## Data Availability

The datasets presented in this study can be found in online repositories. The names of the repository/repositories and accession number(s) can be found in the article/Supplementary Material.
