# Supplementary material for: A rare variation of ERCC8 gene cause Cockayne syndrome in a Chinese family
Source: Front Genet. 2025 Mar 12;16:1531832. doi: 10.3389/fgene.2025.1531832 (PMC11936978; doi:10.3389/fgene.2025.1531832)
Supplement: Supplementary file 1 [file Table1.docx]

Supplementary Material

# Supplementary Figures and Tables

## Supplementary Figures


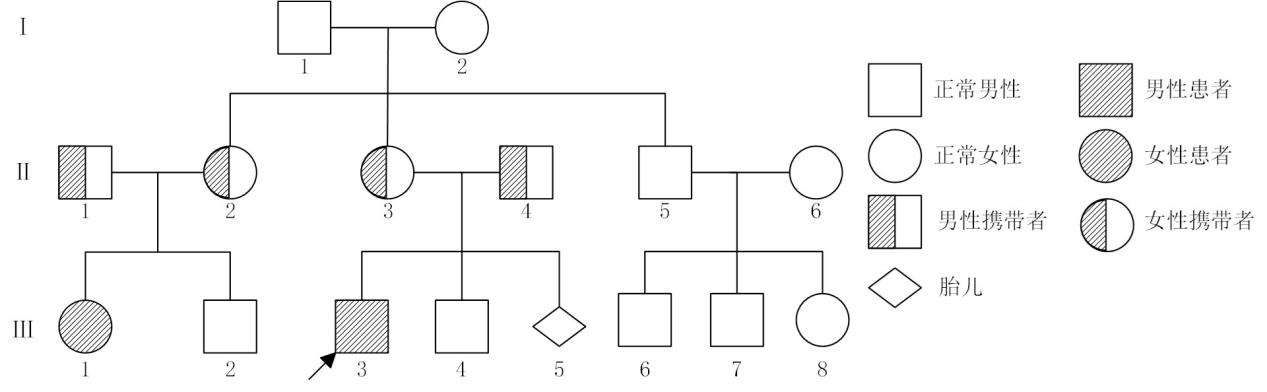


**Supplementary Figure 1.** Pedigree chart showed individuals with or without Cockayne syndrome. Family members II1, II2, II3,II4, III1, III3 and III5 underwent testing. Note: I, II, and III represent the generations. Squares represent males, large circles represent females, solid black symbols indicate affected patients,half-solid black symbols indicate carriers,the arrow indicates the proband.


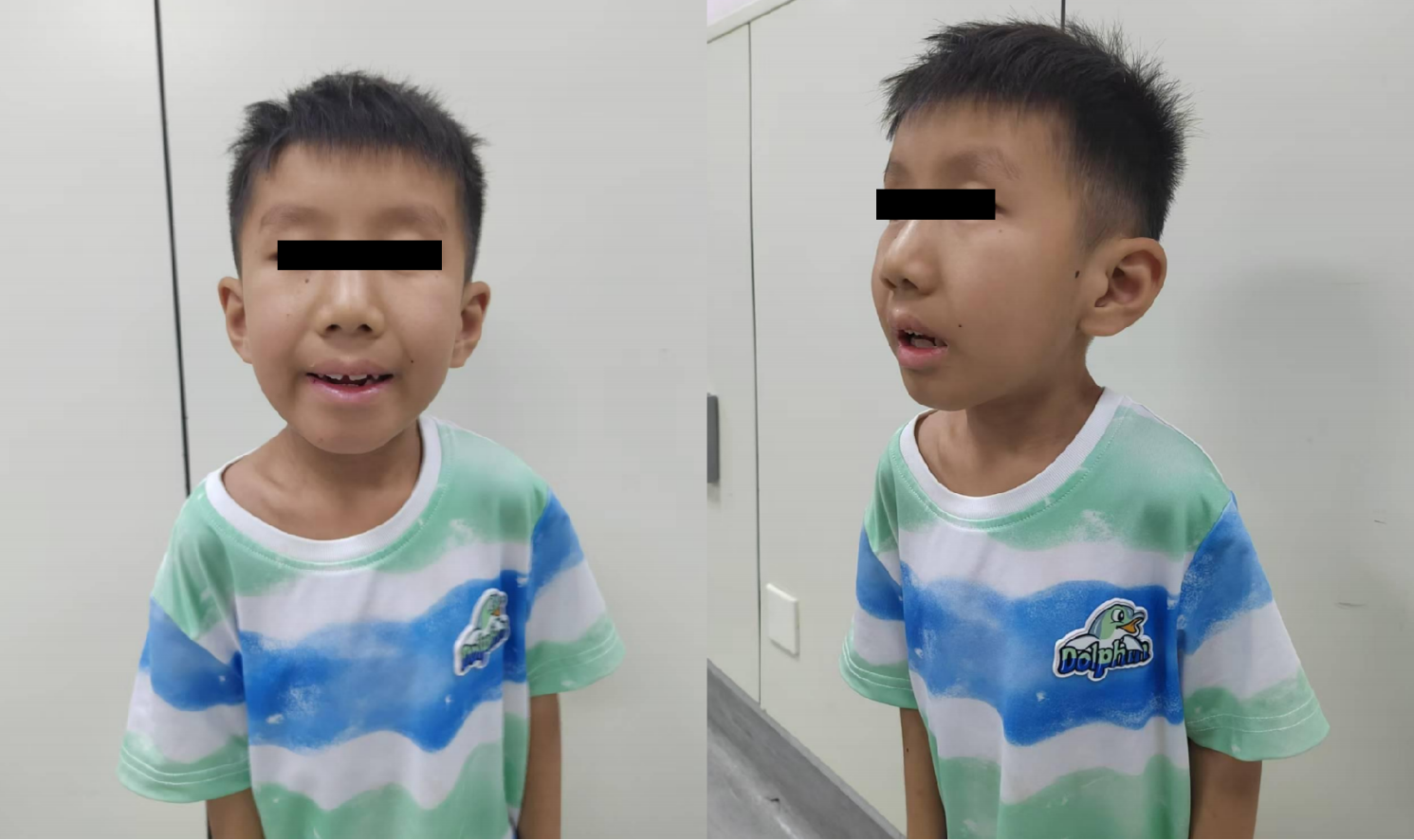


**Supplementary Figure 2**:Clinical characteristics of the proband(III3).


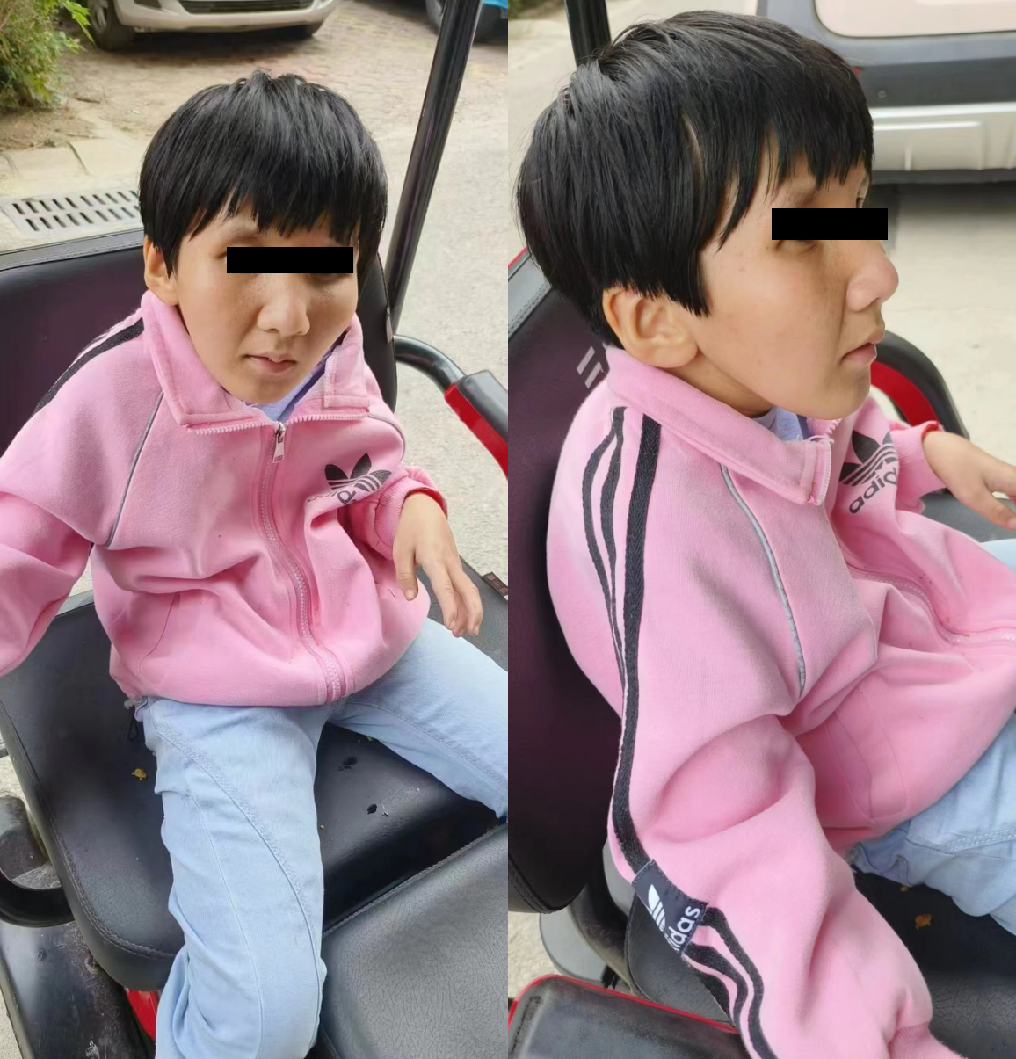


**Supplementary Figure 3**: Clinical characteristics of the patient(III1)


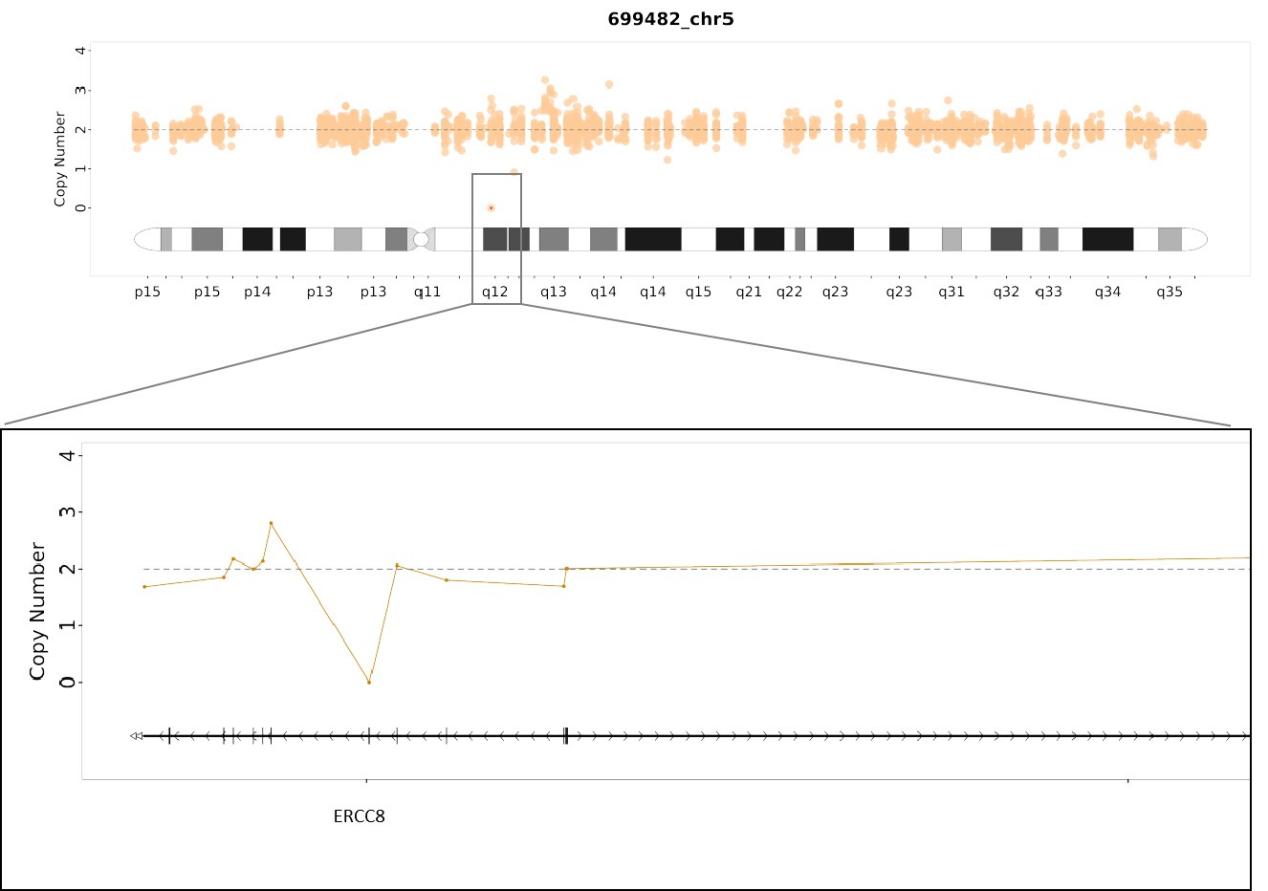


**Supplementary Figure 4**: Medical exome sequecing showed a homozygous deletion of Exon4 in ERCC8 gene of the proband.


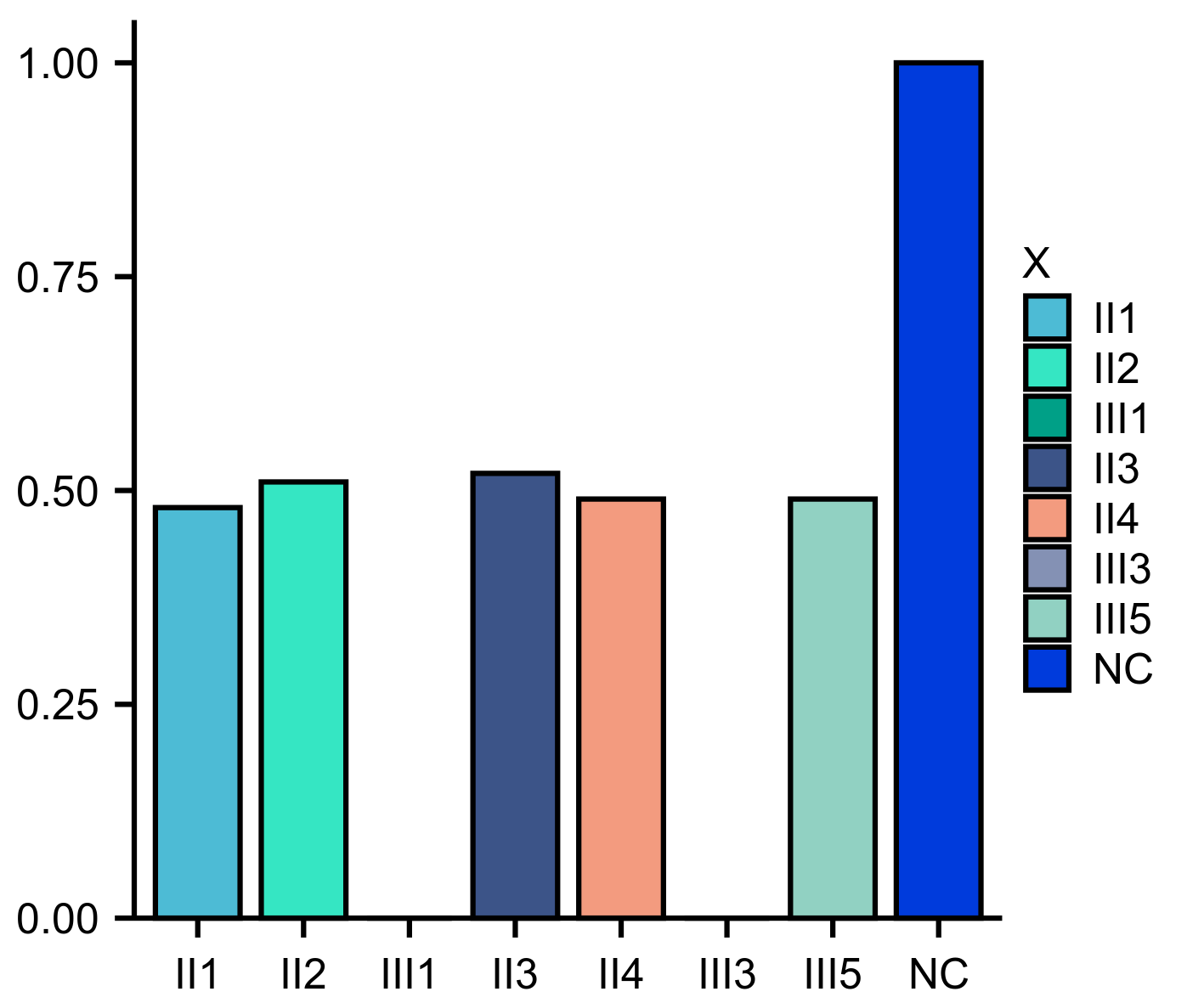


**Supplementary Figure 5**:Family members qPCR verification chart.III1 and III3 copy number is 0.


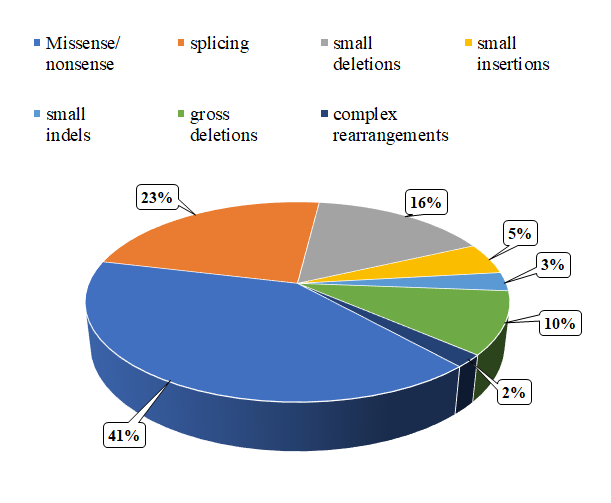


**Supplementary Figure 6：** Distribution of the mutation spectrum in ERCC8 gene reported in HGMD

A total of 99 mutations in ERCC8 gene including 41% missense/nonsense mutations, 23% splicing, 16% small deletions, 5% small insertion, 3% small indels, and 10% gross deletions, and 2% complex rearrangements. The mutations in our patients were all documented by HGMD.

## Supplementary **Table**

**Supplementary Table 1：**Clinical information for the two patients.

| Patient ID | III3 | III1 |
| --- | --- | --- |
| Gender | Male | Female |
| Age of diagnosis | 7 | 15 |
| Growth parameters | | |
| Height | -3SD | -3SD |
| Weight | -3SD | -3SD |
| Facial features | | |
| Microcephaly | -3SD | -3SD |
| Sunken eyes | + | + |
| Pointed nose | + | + |
| Micrognathia | + | + |
| Large auricle | + | + |
| Dental caries | + | + |
| Photosensitivity | + | + |
| Mental retardation | + | + |
| Kyphosis | + | + |
| Restricted motion of joints | + | + |
| Hearing loss | + | + |
| Leukodystrophy | + | unknown |
